# Genomic breeding value prediction and accuracy assessment for carcass traits in Jeju Black Cattle using Hanwoo reference populations

**DOI:** 10.5713/ab.260150

**Published:** 2026-05-18

**Authors:** Md Azizul Haque, Ji-Hee Jang, Monira Akter Mou, Yeeun Kang, Jong-Joo Kim

**Affiliations:** 1 Department of Biotechnology, Yeungnam University, Gyeongsan, Korea; 2 Department of Health Informatics, Faculty of Medicine and Health Sciences, Frontier University, Garowe Campus, Garowe, Somalia

**Keywords:** Accuracy, Breeding Value, Carcass Traits, Genomic Selection, Jeju Black Cattle

## Abstract

**Objective:**

This study aimed to evaluate the accuracy of genomic prediction for carcass traits, specifically carcass weight (CWT), eye muscle area (EMA), backfat thickness (BF), and marbling score (MS) in Jeju Black Cattle (JBC) using a single-trait animal model and to compare the results with those obtained using Korean Hanwoo reference populations.

**Methods:**

The dataset comprised genotypes and phenotypes from 19,153 Hanwoo steers, 6,200 Hanwoo cows, 676 JBC steers, and 654 JBC cows, genotyped using Illumina 50K and Affymetrix 160K SNP chips. The entire population was divided into three groups. In Group 1, Hanwoo steers served as the reference population, whereas JBC steers and cows as the testing population (HSJB). Group 2 expanded the reference population by including both Hanwoo steers and cows, with the same JBC testing set (HAJB). In Group 3, JBC were used only as the reference population (JBJB). The single-trait genomic best linear unbiased prediction analysis was performed using ASReml SA-4.2 software.

**Results:**

In HSJB, theoretical accuracies (ACC_T_) ranged from 0.47 to 0.53, while realized accuracies (ACC_R_) for the total population were 0.23 (CWT), 0.22 (EMA), 0.28 (BF), and 0.53 (MS). For JBC, ACC_R_ ranged from 0.12 to 0.54 in cows and from 0.28 to 0.58 in steers. In Group 2, ACC_T_ improved to 0.48–0.55, with ACC_R_ of 0.25 (CWT), 0.37 (EMA), 0.28 (BF), and 0.47 (MS) in the total population. JBC cows had values between 0.27 to 0.31, while steers from 0.26 to 0.51 across various traits. JBJB showed the highest ACC_T_ values of 0.50–0.54, with ACC_R_ values of 0.46 (CWT), 0.65 (EMA), 0.38 (BF), and 0.55 (MS).

**Conclusion:**

The results indicate that the highest accuracies were obtained in Group 3, where JBC used only as a reference population. Therefore, to increase the prediction accuracy of JBC, it is essential to have breed-specific large populations.

## INTRODUCTION

Jeju Black Cattle (JBC) is one of the indigenous Korean cattle breeds that has been raised for centuries on Jeju Island [1]. This breed is characterized by its unique black coat, compact body size, and strong adaptability to harsh environmental conditions. It possesses unique genetic and cultural value that sets it apart from the more widely recognized Korean Hanwoo cattle [2]. Historically, JBCs were commonly used for draft and agricultural purposes as well as for their beef. However, their population drastically declined due to their slow growth rate and the national focus on improving Hanwoo for commercial beef production [3]. As a result, this indigenous breed was largely ignored by both the government and farmers for genetic improvement. By the late 20th century, the population of JBC had decreased to critically low levels, placing the breed on the edge of extinction. Conservation efforts began following the identification of a small number of remaining animals in 1992 from isolated farms on Jeju and nearby islands [4]. Since then, structured breeding and conservation programs have been implemented to support population recovery and maintain genetic diversity. The Food and Agriculture Organization (FAO) officially recognized JBC as a distinct Korean cattle breed in 2004. In 2013, the Korean government designated JBC as a Natural Monument (No. 546). It also acknowledges its importance as a genetic resource and as part of the nation’s cultural heritage [5].

Despite these conservation efforts, the practical use of JBC for economically important traits such as carcass yield and meat quality remains limited. In contrast, Hanwoo has undergone continuous genetic improvement since the 1960s through coordinated programs led by government and research institutions, with selection focused on productivity and consumer-driven meat quality traits [6]. On the other hand, JBC has not yet received the same level of genetic improvement effort. Most breeding decisions for JBC are still based on visual traits, limited pedigree information, and small population data, which inhibits accurate and effective selection for desirable characteristics [3,7]. Considering the limited population size and restricted genetic diversity of JBC, more precise and evidence-based approaches are needed. These should aim to improve performance while maintaining genetic resources. Environmental conditions also play a significant role. On Jeju Island, the subtropical maritime climate leads to higher temperatures and humidity, which can negatively affect early growth traits such as birth weight and average daily gain. These effects may extend to carcass weight (CWT) and meat quality in later stages [8]. Such environmental variation makes it difficult to separate genetic potential from external influences. This highlights the need for genomic approaches that can more effectively partition genetic and non-genetic sources of variation in JBC.

Genomic selection (GS) provides an effective framework to accelerate genetic gain while controlling inbreeding, which is particularly relevant for small or conserved populations such as JBC. It uses genome-wide molecular markers, most often single nucleotide polymorphisms (SNPs), to estimate the genetic potential of animals at an early stage. This approach reduces reliance on phenotypic records and complete pedigree information [9]. The genomic best linear unbiased prediction (GBLUP) method has emerged as a widely accepted approach in GS, where the traditional pedigree-based numerator relationship matrix (A matrix) is replaced by a genomic relationship matrix (GRM; G matrix) constructed from dense SNP genotype data [10]. This allows for more accurate estimation of genomic estimated breeding values (GEBVs), especially in breeds with limited population sizes or incomplete pedigree records [11]. While GS has significantly transformed breeding programs in commercial cattle breeds such as Holstein and Hanwoo, its implementation in JBC remains at an early stage.

To date, few studies have been conducted to evaluate the genomic architecture or predict GEBVs for carcass traits in JBC [3,11]. Furthermore, there is a lack of comprehensive studies assessing the accuracy of genomic predictions in this breed, which is essential for developing effective breeding programs. The limited reference population and sparse genomic information available for JBC pose additional challenges to implementing GS.

One well-established solution to improve genomic prediction accuracy in breeds with small populations is to incorporate a larger, genetically related breed into the reference population [12,13]. In the present study, Hanwoo was selected as the reference population for three main reasons. First, JBC and Hanwoo share common ancestral origins as native Korean cattle breeds. This shared ancestry suggests a degree of genetic relatedness, which may allow marker-trait associations established in Hanwoo to remain informative for JBC [2]. Second, Hanwoo has a well-documented cattle population in Korea, supported by a national recording system with extensive phenotypic and genotypic data for the carcass traits examined in this study [14]. Third, previous studies have shown that using a larger, genetically related breed as a reference population can substantially improve GEBV accuracy in small or endangered breeds, especially when the target breed lacks a sufficiently large reference population of its own [12]. Based on these considerations, a multi-breed reference population approach was adopted. This allowed us to evaluate whether including Hanwoo data could improve genomic prediction accuracy for JBC carcass traits, while also comparing it against a reference population composed solely of JBC animals.

To address these critical gaps, the present study aims to estimate GEBVs for carcass traits in JBC utilizing high-density whole-genome SNP chip data and to rigorously evaluate the accuracy of genomic predictions across different reference populations using a single-trait animal model. Specifically, this research seeks to develop an optimized reference population by integrating existing phenotypic and genotypic datasets, estimate GEBVs for key carcass traits including CWT, eye muscle area (EMA), backfat thickness (BF), and marbling score (MS), and assess the predictive performance of genomic models for different reference populations. Taken together, these objectives aim to build a practical foundation for implementing GS in JBC. Such a framework could support long-term conservation efforts, improve meat quality and productivity, and strengthen the economic viability of Jeju’s local livestock industry. Ultimately, this work addresses a clear gap in genomic research on this endangered native breed, with direct implications for selection decisions and long-term breeding management. It also contributes to preserving Korea’s agricultural biodiversity by applying genomic tools to a historically neglected population.

## MATERIALS AND METHODS

### Phenotypic data

The phenotypic data from 19,153 Hanwoo steers were collected from different commercial farms across Korea. These steers were slaughtered from 2017 to 2022 at different slaughterhouses throughout the country, with slaughter age ranging from 24 to 35 months. In addition, phenotypic records for 6,200 Hanwoo cows were also collected from various commercial farms that were slaughtered between 2018 and 2024, with ages ranging from 24 to 161 months. Phenotypic records for 676 JBC steers and 654 JBC cows were also collected, which were all raised in the Jeju Special Self-governing Province in Korea. The JBC steers were slaughtered from 2014 to 2024, with the age ranging from 29 to 59 months. On the other hand, JBC cows were slaughtered over the same period at ages ranging between 24 and 143 months. JBC animals were slaughtered in different slaughterhouses in Jeju Special Self-governing Province. The studied traits include CWT, EMA, BF, and MS. The data was collected according to the official Korean carcass grading protocol prepared by the National Livestock Cooperatives Federation. CWT was recorded 24 hours postmortem following refrigeration. EMA was measured using the dot-grid method on a cross-section between the 13th rib and the first lumbar vertebra, where BF was also recorded. CWT, EMA, and BF were measured in kilograms, square centimeters, and millimeters, respectively. MS was assessed visually using a 9-point scale, with a score of 1 indicating no marbling and 9 indicating very high marbling, following the Korean Livestock Products Grading Guidelines.

### Genotyping and quality control

A total of 19,153 Hanwoo steers and 6,200 Hanwoo cows were genotyped using the Illumina BovineSNP50K Chip (Illumina), which includes 53,886 SNPs. In addition, 676 JBC steers and 654 JBC cows were genotyped using a customized Affymetrix 160K SNP Axiom array containing 161,952 SNPs. For quality control (QC), SNPs located on sex chromosomes, as well as those in duplicated or ambiguous genomic positions were removed. A total of 23,915 SNPs that were common between the 50K and customized 160K SNP arrays were retained for analysis. Further QC filtering was applied to eliminate low-quality SNPs. Specifically, SNPs were excluded if their call rate was below 90%, if individuals had a genotyping call rate below 90%, or if the minor allele frequency (MAF) was less than 1% (monomorphic). SNPs that significantly deviated from the Hardy-Weinberg Equilibrium (HWE) (p< 0.000001) were also excluded. An identity-by-state (IBS) test was conducted to detect duplicate individuals or potential genotyping errors. Pairs of individuals with a genetic similarity rate higher than 99% were indicated either as identical animals or genotypic errors. The entire QC procedures, including the IBS test were performed using the PLINK 2.00 alpha tools [15]. In addition, missing alleles were imputed by using the Beagle v5.5 software [16]. After completing IBS and QC filtering, 18,625 Hanwoo steers, 5,989 Hanwoo cows, 656 JBC steers, and 608 JBC cows with genotypes of 18,290 common SNPs remained for further analysis.

### Linkage disequilibrium analysis

Linkage disequilibrium (LD) decay was estimated separately for Hanwoo and JBC populations using PLINK v1.9 [15]. For each population, pairwise r^2^ values were calculated between all SNP pairs within a maximum physical distance of 1,000 kb. LD decay curves were plotted as mean r^2^ against physical distance (kb) to compare the rate of LD decay between Hanwoo and JBC.

### Statistical analysis

The entire population was divided into three groups for genomic prediction. The first group used 18,625 Hanwoo steers as the reference (training) population, while 656 JBC steers and 608 JBC cows were designated as the testing population. The second group expanded the reference population to include 5,989 Hanwoo cows in addition to the 18,625 Hanwoo steers, with the same JBC animals used for testing. In the third group, the reference population consisted solely of the JBC population (656 steers and 608 cows). The same statistical methods were employed across all groups to estimate variance components and evaluate the accuracy of GEBVs.

### Genomic best linear unbiased prediction

The analysis was performed using ASReml SA-4.2 software [17], applying a single-trait GBLUP model as follows:


(1) y=Xb+Zu+e

where, y is the vector of phenotypic values; X is the incidence matrix for fixed effects (b), which includes the farm where the animal was raised, year and season of birth, year and season of slaughter, and slaughter age as a covariate; Z is the design matrix for the random genetic effects (u); u is the vector of individual additive genetic effects, assumed to follow a normal distribution $\text{u}∼\text{N}(0,\text{G}\sigma_{\text{g}}^{2})$; and e is the vector of random residuals, assumed to follow a normal distribution $\text{e}∼\text{N}(0,\text{I}\sigma_{\text{g}}^{2})$. Additionally, $\text{var}(\text{u})=\text{G}\sigma_{\text{u}}^{2}$, where $\sigma_{\text{u}}^{2}$ represents the genetic variance, and G is the GRM, which was constructed as previously described by [18].

### Estimation of genetic parameters

The same dataset and model used in the GBLUP method were applied to estimate the variance components using ASReml SA-4.2 software [17]. Total phenotypic variance was calculated using the formula:


(2) $$\sigma_{\text{p}}^{2}=\sigma_{\text{a}}^{2}+\sigma_{\text{e}}^{2}$$

Heritability (h^2^) for each trait was calculated as:


(3) $${\text{h}}^{2}=\frac{\sigma_{\text{a}}^{2}}{\sigma_{\text{a}}^{2}+\sigma_{\text{e}}^{2}}$$

where $\sigma_{\text{a}}^{2}$ represents the additive genetic variance, $\sigma_{\text{e}}^{2}$ is the residual variance, and $\sigma_{\text{p}}^{2}$ denotes the phenotypic variance.

### Determination of the accuracy

ACC_T_ for GEBV was computed using prediction error variances (PEV) with the following formula [19]:


(4) $${\text{acc}}_{\text{j}}=\sqrt{1-\frac{{\text{PEV}}_{\text{j}}}{\left(1+{\text{F}}_{\text{j}}\right)\sigma_{\text{u}}^{2}}}$$

Where acc_j_ is the accuracy for individual j, PEV represents the prediction error variance, which is calculated as the square of the standard error of prediction for animal j, derived from the inverse of the left-hand side matrix of the mixed model equations, F_j_ stands for the inbreeding coefficient of animal j, and $\sigma_{\text{u}}^{2}$ represents the additive genetic variance.

To evaluate the ACC_R_ of genomic predictions, a five (5) fold cross-validation (CV) method was applied. The entire population in each group (Hanwoo steers as reference and JBC as test [HSJB], Hanwoo steers and cows as reference, JBC as test [HAJB], and JBC as both reference and test population [JBJB]) was randomly divided into five folds. In each round, one fold used as the testing set while the remaining four folds served as the reference set. This random splitting procedure was repeated five times, ensuring that each fold served as the testing set once. The primary objective of this CV method was to reduce sampling errors [20]. The prediction accuracy in each fold was then calculated by using Pearson’s correlation coefficient (r) between the phenotypes of individuals in the testing set and their GEBV, divided by the square root of the heritability for each trait. The empirical standard error (SE) was determined by dividing the standard deviation (SD) of the five calculated accuracies from the fivefold CV by the square root of 5.

## RESULTS

### Description of phenotypes

The summary statistics of carcass traits for Hanwoo and JBC cattle are presented in Table 1. Hanwoo steers exhibited the highest CWT, with an average of 444.41 kg, ranging from 296 to 594 kg. In contrast, JBC steers showed a lower mean CWT of 400.89 kg (range: 249–569 kg). Among cows, Hanwoo also outperformed JBC, with average weights of 371.96 kg and 320.92 kg, respectively. Notably, the difference between steers and cows was more pronounced in the Hanwoo population. In terms of EMA, Hanwoo steers again led with an average of 96.59 cm^2^, followed by Hanwoo cows at 88.03 cm^2^. JBC steers and cows had smaller muscle areas, averaging 79.53 cm^2^ and 76.25 cm^2^, respectively. Interestingly, BF presented a contrasting pattern. JBC cows showed the greatest backfat accumulation, with a mean value of 17.26 mm, followed by JBC steers at 15.86 mm. Meanwhile, Hanwoo steers and cows had lower averages of 13.96 mm and 12.82 mm, respectively. When comparing MS, Hanwoo steers had the highest average score of 5.99. This was substantially greater than that of JBC steers (4.38), Hanwoo cows (4.15), and JBC cows (2.96).

### Description of single nucleotide polymorphism statistics

**Figure 1:**
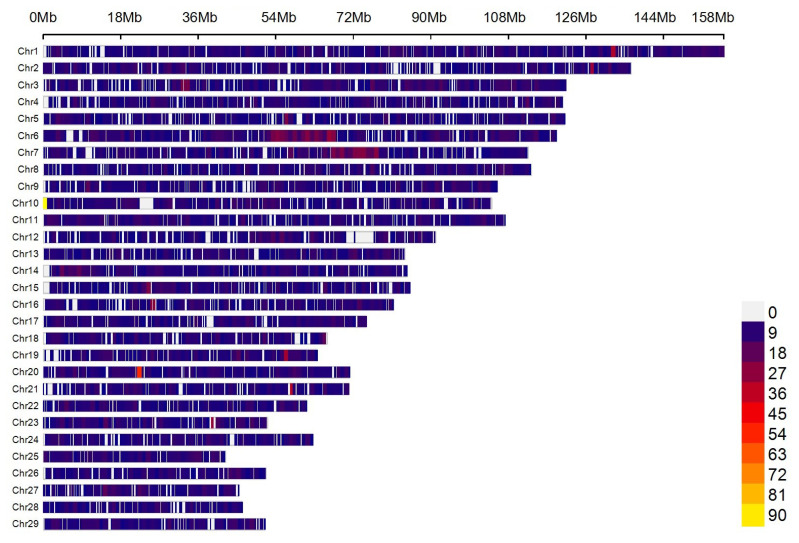
SNP density plot showing the number of SNPs within a 1 Mb window size. The horizontal axis represents the chromosome length (Mb), and the different colors indicate SNP density. SNP, single nucleotide polymorphism.

After performing QC, 18,290 common SNPs were retained across all 29 *Bos taurus* autosomes (BTA). The SNPs were not evenly distributed across the chromosomes. Some chromosomes contained a noticeably higher number of markers. BTA1 had the highest number of SNPs (1,171), spanning approximately 157 Mb, while BTA28 contained the fewest (305 SNPs), over roughly 46.1 Mb (Figure 1). Additionally, BTA1, BTA2, BTA3, and BTA6 each had more than 900 SNPs, indicating higher marker density on these chromosomes.

### Linkage disequilibrium decay

**Figure 2:**
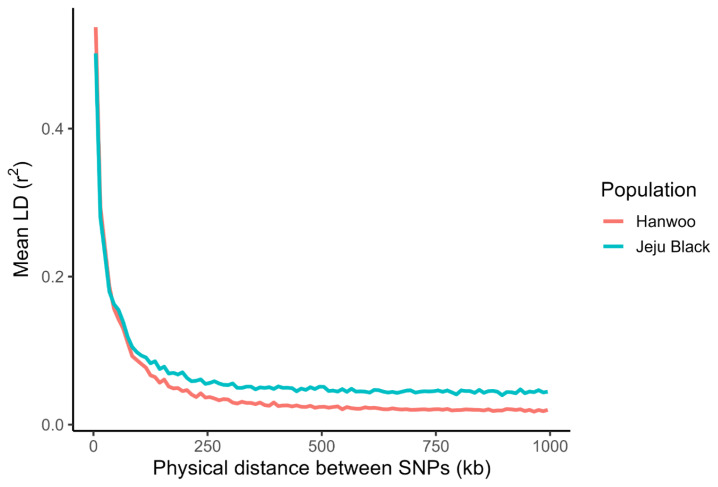
Linkage disequilibrium (LD) decay in Hanwoo and Jeju Black Cattle populations. The x-axis shows physical distance between SNP pairs (kb) and the y-axis shows mean r^2^. SNP, single nucleotide polymorphism.

The LD decay patterns for Hanwoo and JBC populations are shown in Figure 2. In both populations, mean (r^2^) decreased as the physical distance between SNP pairs increased, which is consistent with expected LD. At short distances (10 kb), mean (r^2^) values were 0.391 in Hanwoo and 0.370 in JBC, indicating relatively strong LD at close genomic proximity in both breeds. With increasing distance, LD declined in both populations. At 50 kb, mean (r^2^) was 0.146 in Hanwoo and 0.158 in JBC. At 100 kb, the values further decreased to 0.085 and 0.096, respectively. At 500 kb, mean (r^2^) reached 0.025 in Hanwoo and 0.049 in JBC, reflecting substantial decay at longer distances. The rate of LD decay showed both similarities and differences between the two populations. Both reached the threshold of (r^2^<0.2) at 34.82 kb. However, differences appeared at the (r^2^<0.1) level. Hanwoo reached this threshold at 84.94 kb, whereas JBC required a longer distance of 94.99 kb. The mean (r^2^) across all SNP pairs was 0.045 in Hanwoo and 0.065 in JBC. This further indicates higher background LD in JBC.

### Estimation of heritability

The variance components and heritability estimates for carcass quality traits varied across the groups (Table 2). For HSJB, the highest heritability was observed for MS at 0.44, followed by CWT at 0.39. EMA and BF showed slightly lower heritability estimates of 0.34 and 0.33, respectively. Heritability estimates decreased slightly across all traits in HAJB. CWT showed a heritability of 0.34, while EMA, BF, and MS had values of 0.29, 0.30, and 0.40, respectively. For JBJB, heritability estimates for MS remained consistent with HSJB at 0.44. CWT showed a moderate heritability of 0.36, slightly higher than in HAJB but lower than in HSJB. EMA and BF had heritability estimates of 0.31 and 0.32, respectively. HSJB represents the highest heritability estimates for most traits, particularly for CWT and EMA. JBJB, despite using only JBC animals, maintained competitive heritability values, especially for MS and BF.

### Estimation of genomic estimated breeding value

The results for the GEBV of carcass quality traits across HSJB, HAJB, and JBJB revealed varying distribution in CWT, EMA, BF, and MS among cows and steers in JBC population (Tables 3–5). For HSJB (Table 3), the overall population displays moderately negative values. The mean CWT GEBV is −17.30 (SD = 15.20), while EMA averages −4.79 (SD = 3.35). BF is negative at −0.03, and MS shows a negative tendency at −0.89 (SD = 0.68). Cows in this group follow a similar pattern, with a slightly lower mean CWT of −17.40 and a smaller SD (14.90). EMA is also more negative at −4.84 (SD = 3.44), while BF (−0.04) and MS (−0.90) show minimal deviation from the total population. Steers show comparable results, with a mean CWT of −17.20 and slightly higher variability (SD = 15.40). Their EMA is −4.74 (SD = 3.26), BF remains almost zero at −0.02, and MS stands at −0.88, again closely aligning with the values observed in cows. A more pronounced decline in GEBVs is observed in HAJB (Table 4). The total population observes a mean CWT of −19.40 (SD = 14.50), and EMA drops further to −5.25 (SD = 3.15). BF shows a small negative value of −0.06. MS is also negative at −0.58, but it shows slight improvement with lower variability (SD = 0.64). Cows within this group present values close to the total population, with CWT at −19.20 and EMA slightly lower at −5.33 (SD = 3.23). BF and MS are recorded at −0.08 and −0.60, respectively. Steers reflect the lowest mean CWT among all groups at −19.60 (SD = 14.60), with EMA at −5.17 (SD = 3.08). BF remains relatively stable at −0.03, while MS is −0.56. In contrast, JBJB shows a clear positive shift across all traits (Table 5). The total population shows a near-zero mean CWT of −0.08, albeit with increased variability (SD = 18.80). EMA and BF are close to zero at −0.01 each, while MS is zero at 0.00 (SD = 0.72). They are the only subgroup to exhibit a positive mean CWT (1.18, SD = 19.10) and EMA (0.15, SD = 3.26), while BF (−0.05) and MS (0.04) remain near zero. Steers in JBJB show slightly negative value, with CWT at −1.24 (SD = 18.40), EMA at −0.17, BF slightly positive at 0.03, and MS nearly zero at −0.03 (SD = 0.70).

JBJB presents the most favorable results overall, with near-zero values for all traits, especially EMA and MS. This contrasts with HAJB, where CWT and EMA show more negative, particularly in the steer population. HSJB shows a mix of moderate negative results in CWT and EMA, but with higher variability in BF and MS. Across all groups, the variability is highest in JBJB.

**Figure 3:**
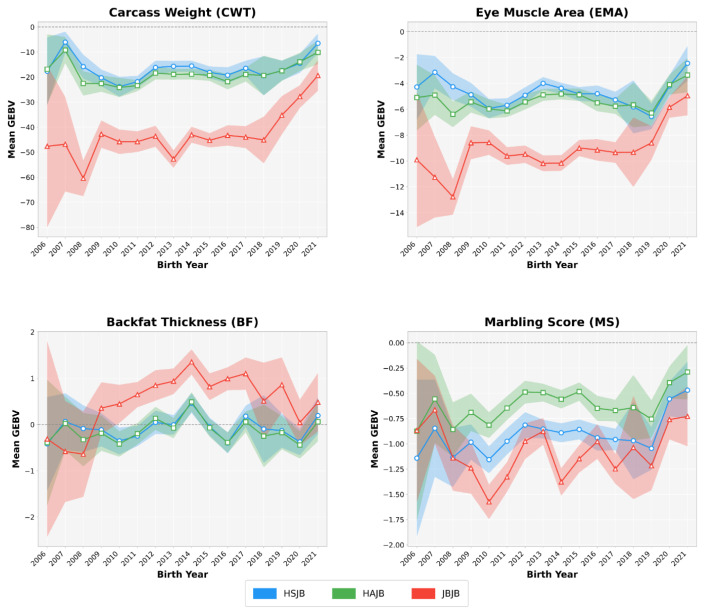
Yearly GEBV trend of Jeju Black Cattle (JBC) carcass trait (mean±95% CI). Each panel shows one trait: carcass weight (CWT), eye muscle area (EMA), backfat thickness (BF), and marbling score (MS). Lines represent annual mean GEBV for HSJB (blue), HAJB (green), and JBJB (red), with shaded ribbons indicating 95% confidence intervals. GEBV, genomic estimated breeding value; HSJB, Hanwoo steers as reference and JBC as test; HAJB, Hanwoo steers and cows as reference, JBC as test; JBJB, JBC as both reference and test population.

Additionally, Figure 3 shows the yearly trend of GEBV for all carcass traits across the three groups. For CWT and EMA, JBJB generally maintains higher mean GEBV over time compared to HSJB and HAJB. BF shows more fluctuation across years, with no consistent trend among groups. For MS, JBJB shows a gradual increase, while HSJB and HAJB remain relatively stable. The confidence intervals overlap in several years, but JBJB often shows higher mean values, especially for MS.

### Evaluation of genomic estimated breeding value prediction accuracy

Genomic prediction accuracy varied across traits and reference population structures (Tables 6, 7). The ACC_T_ showed only slight variation across groups, while the ACC_R_ showed clear differences.

For CWT, the ACC_T_ values were almost similar across groups (Table 6). For all JBC individuals, ACC_T_ was 0.51±0.02 in HSJB, 0.52±0.02 in HAJB, and 0.50±0.02 in JBJB. Among JBC cows, the values were slightly higher, with 0.52±0.03 (HSJB), 0.53±0.02 (HAJB), and 0.51±0.02 (JBJB). JBC steers showed comparable values, ranging from 0.50±0.03 to 0.52±0.03. However, ACC_R_ values showed clear differences across groups (Table 7). In the all JBC individuals, ACC_R_ increased from 0.23±0.03 (HSJB) to 0.25±0.03 (HAJB) and reached 0.46±0.03 in JBJB. Among JBC cows, ACC_R_ improved from 0.21±0.03 (HSJB) to 0.28±0.03 (HAJB) and 0.63±0.03 (JBJB). A similar pattern was observed in JBC steers, where ACC_R_ increased from 0.33±0.03 to 0.39±0.03 and then to 0.71±0.03.

A different pattern was observed for EMA. Although ACC_T_ values remained stable across groups (0.46–0.48 in JBC individuals), ACC_R_ showed the notable improvement among all traits (Table 7). In the all JBC population, ACC_R_ increased from 0.22±0.03 (HSJB) to 0.37±0.03 (HAJB) and reached 0.65±0.03 in JBJB. Among JBC cows, EMA accuracy increased from 0.12±0.03 to 0.27±0.03 and then to 0.63±0.03. For JBC steers, ACCR increased from 0.33±0.03 (HSJB) to 0.46±0.03 (HAJB) and further to 0.73±0.03 in JBJB.

For BF, both ACC_T_ and ACC_R_ showed comparatively smaller variation. ACC_T_ values were ranging from 0.46±0.03 to 0.49±0.03 in the all JBC population (Table 6). Among JBC cows, values ranged from 0.48±0.03 to 0.50±0.03, while JBC steers showed slightly higher values in HAJB and JBJB (0.50–0.51±0.03). The ACC_R_ results showed slight improvement (Table 7). In the all JBC individuals, ACC_R_ remained similar between HSJB and HAJB (0.28±0.03) and increased to 0.38±0.03 in JBJB. Among JBC cows, ACC_R_ improved from 0.30±0.03 (HSJB) to 0.31±0.03 (HAJB) and 0.40±0.03 (JBJB). For JBC steers, values were relatively stable in HSJB and HAJB (0.28±0.03 and 0.26±0.03), but increased to 0.37±0.03 in JBJB.

In contrast, MS maintained consistently high ACC_T_ values across all groups, ranging from 0.53±0.02 to 0.55±0.04 (Table 6). The ACC_R_ values also showed relatively strong performance compared to other traits. In the all JBC population, ACC_R_ was 0.53±0.03 in HSJB, slightly decreased to 0.47±0.03 in HAJB, and increased again to 0.55±0.03 in JBJB (Table 7). Notably, JBC steers showed a clear improvement, reaching 0.66±0.03, while JBC cows also showed an increase to 0.58± 0.03.

### Genetic relatedness

The genetic relatedness between Hanwoo and JBC populations was assessed using the GRM (G-matrix). The average genetic relationship among Hanwoo individuals was estimated to be 0.0004212. In contrast, the average genetic relationship between Hanwoo and JBC individuals was found to be −0.0089897. Meanwhile, the average genetic relationship among JBC individuals was substantially higher, with a value of 0.1743447.

## DISCUSSION

The estimates of heritability for carcass traits in JBC differ across reference population composition. When Hanwoo steers alone were used as the reference, the heritability estimates were generally higher compared to those obtained by combining Hanwoo steers and cows or using only JBC animals. This suggests that using a more genetically homogeneous reference population may increase the accuracy of genetic parameter estimation, likely due to reduced environmental heterogeneity [21]. The slight reduction observed in heritability when Hanwoo cows were included in the reference population indicates that adding animals raised under different management or environmental conditions can inflate residual variance and weaken the genetic signal [22]. Since steers and cows are typically raised under different production systems and face distinct physiological demands, their inclusion can introduce additional environmental variation. This added variance may capture the detection of additive genetic variance effects, ultimately lowering the accuracy of heritability estimates. This finding highlights the importance of carefully considering animal classes when expanding reference populations, even within the same breed. The heritability estimates in this study ranged from low to moderate, which aligns with previously reported values for the same breed. For instance, Haque et al. reported estimates of 0.24 to 0.40 for carcass traits in JBC [11]. Another study estimated the heritability values of 0.42 for CWT, 0.27 for EMA, 0.26 for BF, and 0.48 for MS [3].

Although there are limited reports specifically on JBC carcass traits heritability, comparisons could be drawn with Korean Hanwoo and other cattle populations. In Hanwoo, moderate heritability estimates have been observed across various genomic evaluation methods, including GBLUP (0.30–0.39), BayesA (0.33–0.41), BayesB (0.29–0.36), BayesC (0.31–0.38), and BayesCPi (0.32–0.39), as reported by [23]. In contrast, Alam et al. [24] reported higher heritability values using traditional pedigree and phenotype-based models in Hanwoo steers. Similarly, Mehrban et al. [25], analyzing data from 590 Hanwoo young bulls, found heritability estimates of 0.31 for CWT, 0.43 for EMA, 0.49 for BF, and 0.61 for MS. Moreover, Bhuiyan et al. found heritability values of 0.51 for CWT, 0.45 for EMA, 0.29 for BF, and 0.22 for MS in Hanwoo slaughtered at or under 30 months of age [26]. This difference is expected, as heritability estimates based on genomic data, especially those using SNP markers, are often lower than those calculated from pedigree-based models. This observation is consistent with findings by Yang et al. [27]. Notably, the JBC-only reference group maintained relatively stable heritability estimates, particularly for MS and BF. This finding indicates the potential of breed-specific reference populations, even those of smaller size or with limited diversity, to generate reliable genetic parameter estimates for economically important traits. Moreover, the consistently high heritability of MS across all groups underscores the strong genetic control of this trait. This stability suggests that MS can be a key selection target for JBC breeding programs, with the potential to respond well to GS regardless of reference population composition.

The GEBV estimations for carcass quality traits exhibited distinct variability patterns, emphasizing the importance of reference population composition on genetic predictions. However, the breeding values reported on the carcass traits for the JBC are limited compared to other beef cattle like Hanwoo. A recent study reported GEBVs for the CWT, EMA, BFT, and MS traits as 7.348, 1.515, −0.355, and 0.040, respectively using single-step GBLUP [28]. Importantly, another study using ssGBLUP resulted in significantly higher GEBVs in a Hanwoo full-sib family than our study, where the values for the CWT, EMA, BFT, and MS were 21.5±27.4, 5.5±5, −0.9±1.4, and 0.9±0.8, respectively [29]. A very recent study revealed a wide range of GEBVs for the CWT (−71.8 to 106), EMA ( −19.2 to 22.5), BF (−9.08 to 7.33), and MS (−3.30 to 3.67) traits in Korean Hanwoo cows using GBLUP [30]. In this investigation, HSJB showed negative value in CWT and EMA, with inconsistent fat and marbling quality, indicating a gradual loss in overall carcass size and muscularity on JBC over time. Similarly, HAJB showed larger declines in CWT and EMA. These values decreased further after adding cows to the reference population. This is likely because cows have lower CWT and EMA. Additionally, steers showed higher phenotypic averages, GEBV values were estimated after adjusting for fixed effects such as farm, year, season, and slaughter age. These adjustments likely reduced the observed advantage of steers, resulting in similar or slightly lower mean GEBV values compared to cows. On the other hand, JBJB exhibited the higher GEBV variability across all four traits compared to HSJB and HAJB. This may be because the population had low genetic diversity. It may also be more affected by environmental factors due to a homogeneous reference population. The yearly GEBV trends shown in Figure 3 reveal clear differences among the three groups, highlighting the strong effect of reference population composition on genetic evaluation. JBJB shows a more consistent and slightly positive trend across most traits, especially for MS, while HSJB and HAJB display relatively stable or declining patterns for CWT and EMA. This suggests that within-breed evaluation captures genetic merit more effectively, whereas using different breeds in the reference and test populations may reduce prediction accuracy [31].

GS is extensively applied in the beef industry, especially for improving carcass and meat quality traits [32]. Numerous research efforts have assessed how accurately genomic prediction models perform across various breeds and traits [33]. In Hanwoo steers, the GBLUP method achieved GEBV accuracies between 0.78 and 0.81 for the CWT, EMA, BFT, and MS [34]. On the other hand, Lee et al. [35] reported comparatively lower GEBV accuracies in the Hanwoo population, ranging from 0.54 to 0.78. Another investigation observed GEBV accuracies of 0.7±0.0 for all carcass traits (CWT, EMA, BF, and MS) with ssGBLUP in a Hanwoo full-sib family [29], which partially corresponds with our results. Utilizing 919 Hanwoo cattle, Kim et al. [36] also found higher GBLUP prediction accuracies for CWT (0.779), EMA (0.758), BF (0.766), and MS (0.791), which are partly consistent with our findings.

Our study found ACC_T_ lower than a recent investigation using GBLUP in Hanwoo cows with values of 0.73, 0.68, 0.68, and 0.74 for CWT, EMA, BF, and MS, respectively [37]. Another recent study observed ACC_T_ ranging from 0.75–0.78 for CWT, 0.74–0.76 for EMA, 0.75–0.77 for BF and 0.78–0.81 for MS; and ACC_R_ ranging from 0.70–0.74 for CWT, 0.64–0.69 for EMA, BF 0.59–0.65 and 0.73–0.78 for MS respectively in Hanwoo cows [30], which is significantly higher than our findings. The possible reason for these differences is that their study used within-breed reference and test populations. In contrast, our HSJB and HAJB used different breeds as reference and test populations. Furthermore, lower genomic prediction accuracies for EMA, BF, and MS ranging from 0.27 to 0.30 were also reported in Korean Hanwoo cattle using a 50K SNP chip [38]. This study found that MS had the highest GEBV accuracy while EMA had the lowest, which is probably due to variations in trait heritability [35]. Several studies reported slightly higher ACC_R_ in the Hanwoo population than our findings with a range of 0.43–0.54 for the carcass traits [35,39], which might be because of differences in reference population size and genetic relationships [30]. However, in JBJB, where the JBC population alone served as the reference, the accuracy was higher than that reported by Lee et al. [28] in Hanwoo. This aligns with our observation that MS showed the highest accuracy while EMA had the lowest.

The GEBV prediction accuracy exhibited unique patterns across reference populations. HSJB displayed moderate ACC_T_ due to genetic uniformity, but lower ACC_R_ for all carcass traits, this might be for data quality, population structure, and genetic architecture. Despite a minor reduction in ACC_T_, HAJB exhibited comparatively higher ACC_R_, particularly for CWT and EMA, owing to increased genetic diversity. Among all three groups, JBJB showed the higher accuracy in both ACC_T_ and ACC_R_. This suggests that within-breed evaluation provides more reliable prediction accuracy. The higher genetic relatedness within the JBC population likely contributed to this improved performance.

The LD results further support these findings. Although both Hanwoo and JBC showed similar LD decay at short distances, JBC maintained higher LD at longer distances and showed slower decay at the (r^2^<0.1) threshold. The overall mean r^2^ was also higher in JBC. This suggests more persistent LD in JBC, which may be due to a smaller effective population size or stronger genetic relatedness within the breed. However, even with higher LD, the multibreed prediction accuracy remained low. This may be because LD phase and allele frequencies differ between Hanwoo and JBC. As a result, marker effects estimated in one breed may not be directly applicable to another breed [31].

The genetic relationship within the JBC population was notably high, with a value of 0.1743447 compared to the Hanwoo population (0.0004212), suggesting that JBC are more genetically homogeneous compared to Hanwoo, due to a more closed breeding structure, possibly reflecting a smaller effective population size or strong selection within the JBC line. Furthermore, compared to JBC, the lower value implies that Hanwoo individuals have more genetic diversity and are less linked, which could be the result of different sire contributions or selection procedures that refuse to put a high priority on similarities in genetic makeup in this group [39]. Meanwhile, the genetic relationship between Hanwoo and JBC individuals was negative (−0.0089897), indicating genetic divergence and very limited genetic overlap between the two populations [40]. This low genetic relationship likely contributed to the reduced prediction accuracy observed in HSJB and HAJB. One study [30] found the higher genetic relatedness among cows (0.001277) compared to steers (0.0000919) in the Hanwoo population, which is comparatively lower than our estimations. However, genomic prediction accuracy relies on the genetic closeness between the reference and testing populations. Since the JBC disclosed higher relatedness within the group, using a reference population composed of genetically similar individuals (JBC) can substantially enhance prediction accuracy. This finding reinforces the established principle that GS achieves the highest accuracy when the reference population closely mirrors the genetic makeup of the target population [41]. In contrast, a reference population consisting of genetically distant individuals provides limited benefit to prediction accuracy.

Our results show how the composition of the reference population affects the accuracy of genomic predictions for key economic traits such as CWT, EMA, BF, and MS in the JBC population. However, research on JBC remains limited, and there is a lack of established breeding strategies incorporating GS for this population. The small population size of JBC could reduce the accuracy and robustness of genomic predictions. Additionally, insufficient genetic variation within reference populations may limit the prediction of diverse genetic backgrounds. Despite these challenges, incorporating breed-specific reference data could facilitate the identification of unique genetic variants and trait associations relevant to JBC, thereby improving genomic evaluations in JBC breeding programs.

In this study, a single-trait animal model was applied to each carcass trait separately. Multi-trait models can improve GEBV accuracy by using genetic correlations among traits [42]. The single-trait model provided a more practical and computationally feasible framework. It also improved transparency, as prediction accuracy could be assessed separately for each trait. This avoided potential complications associated with estimating genetic correlations across breeds, which can be unstable when population structures differ considerably. Future studies with larger and more uniform datasets may benefit from adopting a multi-trait framework to further improve prediction accuracy.

From a practical breeding perspective, the effectiveness of a GS program depends not only on prediction accuracy but also on genetic gain per unit time, which integrates selection intensity, generation interval, and accuracy. Although a formal genetic gain analysis was beyond the scope of this study due to the absence of generation interval data specific to JBC, the substantially higher ACC_R_ achieved in JBJB suggests greater potential for genetic gain through within-breed GS. Future studies incorporating generation interval estimates and selection intensity parameters for JBC would enable a more comprehensive comparison of breeding program efficiency across the reference population configurations evaluated here.

## CONCLUSION

This study highlights the critical role of reference population composition in the application of GS for JBC. The outcomes of this study indicate that using the same breed for both the reference and test population, as in JBJB, yields substantially higher prediction both in ACC_T_ and ACC_R_, compared to other reference scenarios. This improvement was particularly evident for EMA and MS, where the ACC_T_ and ACC_R_ were closely aligned, enhancing the reliability of predictions for practical breeding applications. Notably, this breed-specific approach proved more accurate and consistent, reflecting the advantage of capturing genetic variation unique to the target breed. In conclusion, employing a JBC-specific reference population offers a robust and reliable framework for genomic prediction of carcass quality traits in JBC. By leveraging breed-specific genetic information, this strategy can enhance the precision of GS, particularly for traits influenced by unique genetic variability, and ultimately strengthen breeding programs aimed at improving meat quality in JBC.

## Figures and Tables

**Table 1:** Summary statistics for the Hanwoo and JBC carcass quality traits

Population	Traits	N	Minimum	Maximum	Average	SD
Hanwoo steers	CWT (kg)	18,625	296	594	444.41	47.22
	EMA (cm^2^)	18,625	61	133	96.59	11.56
	BF (mm)	18,625	1	28	13.96	4.42
	MS (score)	18,625	1	9	5.99	1.85
Hanwoo cows	CWT (kg)	5,989	214	536	371.96	51.62
	EMA (cm^2^)	5,989	48	128	88.03	12.66
	BF (mm)	5,989	2	30	12.82	5.19
	MS (score)	5,989	1	9	4.15	1.96
JBC steers	CWT (kg)	656	249	569	400.89	53.57
	EMA (cm^2^)	656	52	106	79.53	9.54
	BF (mm)	656	2	34	15.86	5.57
	MS (score)	656	1	9	4.38	1.97
JBC cows	CWT (kg)	608	201	475	320.92	46.78
	EMA (cm^2^)	608	44	106	76.25	10.30
	BF (mm)	608	3	39	17.26	7.02
	MS (score)	608	1	9	2.96	1.78

JBC, Jeju Black Cattle; N, total number of individuals; SD, standard deviation; CWT, carcass weight; EMA, eye muscle area; BF, backfat thickness; MS, marbling score.

**Table 2:** Estimated heritability and partitioning of variances for carcass quality traits

Groups	Traits	$\sigma_{\text{a}}^{2}$	$\sigma_{\text{e}}^{2}$	$\sigma_{\text{p}}^{2}$	h^2^
HSJB	CWT	704.00	1,083.00	1,787.00	0.39
	EMA	38.20	74.80	113.00	0.34
	BF	5.83	11.80	17.70	0.33
	MS	1.29	1.62	2.90	0.44
HAJB	CWT	630.00	1,209.00	1,839.00	0.34
	EMA	34.10	84.00	118.00	0.29
	BF	5.78	13.50	19.30	0.30
	MS	1.17	1.80	2.97	0.40
JBJB	CWT	689.00	1,218.00	1,907.00	0.36
	EMA	25.60	57.60	83.20	0.31
	BF	11.70	24.90	36.60	0.32
	MS	1.30	1.66	2.96	0.44

$\sigma_{\text{a}}^{2}$, genetic variance; $\sigma_{\text{e}}^{2}$, residual variance; $\sigma_{\text{p}}^{2}$, phenotypic variance; HSJB, Hanwoo steers as reference and JBC as test; CWT, carcass weight; EMA, eye muscle area; BF, backfat thickness; MS, marbling score; JBC, Jeju Black Cattle; HAJB, Hanwoo steers and cows as reference, JBC as test; JBJB, JBC as both reference and test population.

**Table 3:** Statistics for GEBV of carcass quality traits in HSJB for JBC

Traits	Minimum	Maximum	Mean	SD
CWT	−71.20	55.40	−17.30	15.20
EMA	−15.10	9.14	−4.79	3.35
BF	−4.24	4.23	−0.03	1.30
MS	−2.71	2.25	−0.89	0.68
CWT	−71.20	55.40	−17.40	14.90
EMA	−13.30	7.56	−4.84	3.44
BF	−4.24	3.95	−0.04	1.34
MS	−2.67	2.13	−0.90	0.69
CWT	−55.90	37.90	−17.20	15.40
EMA	−15.10	9.14	−4.74	3.26
BF	−4.12	4.23	−0.02	1.27
MS	−2.71	2.25	−0.88	0.67

Number of animals in all (cow+steer), cow and steer categories were 1,264, 608, and 656, respectively.

GEBV, genomic estimated breeding value; HSJB, Hanwoo steers as reference and JBC as test; JBC, Jeju Black Cattle; SD, standard deviation; CWT, carcass weight; EMA, eye muscle area; BF, backfat thickness; MS, marbling score.

**Table 4:** Statistics for GEBV of carcass quality traits in HAJB for JBC

Traits	Minimum	Maximum	Mean	SD
CWT	−66.40	45.50	−19.40	14.50
EMA	−15.70	8.43	−5.25	3.15
BF	−4.73	4.78	−0.06	1.32
MS	−2.22	2.44	−0.58	0.64
CWT	−66.40	45.50	−19.20	14.40
EMA	−12.90	7.06	−5.33	3.23
BF	−4.73	4.78	−0.08	1.37
MS	−2.22	2.10	−0.60	0.64
CWT	−61.10	33.70	−19.60	14.60
EMA	−15.70	8.43	−5.17	3.08
BF	−3.84	4.37	−0.03	1.26
MS	−2.12	2.44	−0.56	0.63

Number of animals in all (cow+steer), cow and steer categories were 1,264, 608, and 656, respectively.

GEBV, genomic estimated breeding value; HAJB, Hanwoo steers and cows as reference, JBC as test; JBC, Jeju Black Cattle; SD, standard deviation; CWT, carcass weight; EMA, eye muscle area; BF, backfat thickness; MS, marbling score.

**Table 5:** Statistics for GEBV of carcass quality traits in JBJB for JBC

Traits	Minimum	Maximum	Mean	SD
CWT	−61.20	55.90	−0.08	18.80
EMA	−10.50	10.00	−0.01	3.21
BF	−4.71	3.69	−0.01	1.33
MS	−1.95	2.15	0.00	0.72
CWT	−61.2	53.80	1.18	19.10
EMA	−10.00	9.00	0.15	3.26
BF	−4.71	3.69	−0.05	1.35
MS	−1.57	2.15	0.04	0.74
CWT	−51.20	55.90	−1.24	18.40
EMA	−10.50	10.00	−0.17	3.16
BF	−4.50	3.35	0.03	1.31
MS	−1.95	1.94	−0.03	0.70

Number of animals in all (cow+steer), cow and steer categories were 1,264, 608, and 656, respectively.

GEBV, genomic estimated breeding value; JBJB, JBC as both reference and test population; JBC, Jeju Black Cattle; SD, standard deviation; CWT, carcass weight; EMA, eye muscle area; BF, backfat thickness; MS, marbling score.

**Table 6:** ACC_T_ for carcass quality traits in Jeju Black Cattle (JBC)

Population	Trait	HSJB	HAJB	JBJB
Total	CWT	0.51±0.02	0.52±0.02	0.50±0.02
	EMA	0.47±0.03	0.48±0.03	0.46±0.03
	BF	0.47±0.03	0.49±0.03	0.46±0.03
	MS	0.53±0.02	0.55±0.04	0.53±0.03
Cow	CWT	0.52±0.03	0.53±0.02	0.51±0.02
	EMA	0.48±0.02	0.49±0.03	0.47±0.03
	BF	0.48±0.03	0.50±0.03	0.48±0.03
	MS	0.54±0.02	0.56±0.02	0.54±0.02
Steer	CWT	0.50±0.03	0.52±0.02	0.52±0.03
	EMA	0.46±0.03	0.50±0.03	0.51±0.04
	BF	0.46±0.03	0.50±0.03	0.51±0.03
	MS	0.53±0.02	0.54±0.02	0.54±0.02

HSJB, Hanwoo steers as reference and JBC as test; HAJB, Hanwoo steers and cows as reference, JBC as test; JBJB, JBC as both reference and test population; CWT, carcass weight; EMA, eye muscle area; BF, backfat thickness; MS, marbling score.

**Table 7:** ACC_R_ for carcass quality traits in Jeju Black Cattle (JBC)

Population	Trait	HSJB	HAJB	JBJB
Total	CWT	0.23±0.03	0.25±0.03	0.46±0.03
	EMA	0.22±0.03	0.37±0.03	0.65±0.03
	BF	0.28±0.03	0.28±0.03	0.38±0.03
	MS	0.53±0.03	0.47±0.03	0.55±0.03
Cow	CWT	0.21±0.03	0.28±0.03	0.63±0.03
	EMA	0.12±0.03	0.27±0.03	0.63±0.03
	BF	0.30±0.03	0.31±0.03	0.40±0.03
	MS	0.54±0.03	0.47±0.03	0.58±0.03
Steer	CWT	0.33±0.03	0.39±0.03	0.71±0.03
	EMA	0.33±0.03	0.46±0.03	0.73±0.03
	BF	0.28±0.03	0.26±0.03	0.37±0.03
	MS	0.58±0.03	0.51±0.03	0.66±0.03

HSJB, Hanwoo steers as reference and JBC as test; HAJB, Hanwoo steers and cows as reference, JBC as test; JBJB, JBC as both reference and test population; CWT, carcass weight; EMA, eye muscle area; BF, backfat thickness; MS, marbling score.

## Data Availability

The data used in this study are accessible on Figshare ( https://doi.org/10.6084/m9.figshare.30797732).
